# Thermodynamics of Surfactants, Block Copolymers and Their Mixtures in Water: The Role of the Isothermal Calorimetry

**DOI:** 10.3390/ijms10072873

**Published:** 2009-06-29

**Authors:** Rosario De Lisi, Stefania Milioto, Nicola Muratore

**Affiliations:** Dipartimento di Chimica Fisica “F. Accascina,” Università degli Studi di Palermo, Viale delle Scienze, Parco D’Orleans II, 90128 Palermo, Italy; E-Mails: delisi@unipa.it (R.D.L.); nicola.muratore@unipa.it (N.M.)

**Keywords:** copolymer, surfactant, copolymer+surfactant mixtures, enthalpy of micellization, enthalpy of injection, enthalpy of transfer, enthalpy of copolymer+surfactant aggregation

## Abstract

The thermodynamics of conventional surfactants, block copolymers and their mixtures in water was described to the light of the enthalpy function. The two methodologies, i.e. the van’t Hoff approach and the isothermal calorimetry, used to determine the enthalpy of micellization of pure surfactants and block copolymers were described. The van’t Hoff method was critically discussed. The aqueous copolymer+surfactant mixtures were analyzed by means of the isothermal titration calorimetry and the enthalpy of transfer of the copolymer from the water to the aqueous surfactant solutions. Thermodynamic models were presented to show the procedure to extract straightforward molecular insights from the bulk properties.

## 1. Introduction

Conventional surfactants are amphiphilic molecules with polar head groups, which may be anionic, cationic, non-ionic and zwitterionic, and hydrophobic tails, that may be hydrogenated or fluorinated, linear or branched. Recently, some interest has been devoted to the new class of so-called *gemini* surfactants [1–4]. They are composed of two polar heads flanked by a spacer to which hydrophobic tails are linked; the spacer can be rigid or flexible, polar or apolar.

Attention has been also addressed to the family of polymeric surfactants which are copolymers with two or more blocks having variable monomeric composition. These macromolecules therefore offer the great advantage to be properly tuned by modulating the molecular weight, the composition and the hydrophobic/hydrophilic portions [5].

Finally, a peculiar class of surfactants is represented by the amphiphilic cyclodextrins [6] which possess at the same time the properties of inclusion and of self-organization resulting very promising for an enhanced encapsulation of solutes sparingly soluble in water.

Surfactants are interesting molecules from both the scientific and the industrial view-points because of their characteristics: 1) they are active at the interface (liquid/air, liquid/liquid, solid/liquid, etc.); and 2) at a given surfactant concentration, called critical micellar concentration, they self-assemble into nanostructures called micelles. Therefore, they can be employed to design and create nanostructures functional to various purposes (pharmaceuticals, enhanced oil solubilization, cosmetics, colloidal stabilization, Cultural Heritage, remediation technologies, etc.). Hereafter, a very few examples of their applications are illustrated.

In the field of the restoration and Cultural Heritage conservation, one of the fundamental steps deals with cleaning up the art-work. The conventional cleaning systems are unsafe because they are based on toxic and aggressive organic solvents. Designing cleaning solvents with a low environmental impact and safe for both the art-work and the operator is therefore an important task. The aqueous mixtures of conventional surfactants [7,8] are good cleaning agents of art-works for the following reasons:

1. since the surfactants are active at the interface, they reduce the interfacial tension weakening the attractive forces between the solid substrate and the undesired materials (varnish, pollutants, etc.);
2. the self-organized aggregates are able to increase the solubility of hydrophobic compounds;
3. the interfacial area between the surface of the art-work and the undesired material is very large having surfactant systems a high surface/volume ratio;
4. the micelles are matrices for entrapping organic solvents;
5. surfactant solutions form gels useful from the practical point of view because the penetration by capillarity into the porosities or microfractures of the art-work is strongly reduced, the area to be cleaned is under control and the removal of gels is easy;
6. the amount of surfactant to prepare such systems is relatively low so that the inconveniences related to the eventual residues after the gel removal and the cost of the operation are low;
7. the high sensitivity to temperature of some polymeric surfactants may allow to apply the solvent mixture in the gel phase and to remove it in the fluid state by a slight temperature change.

**Figure 1.**
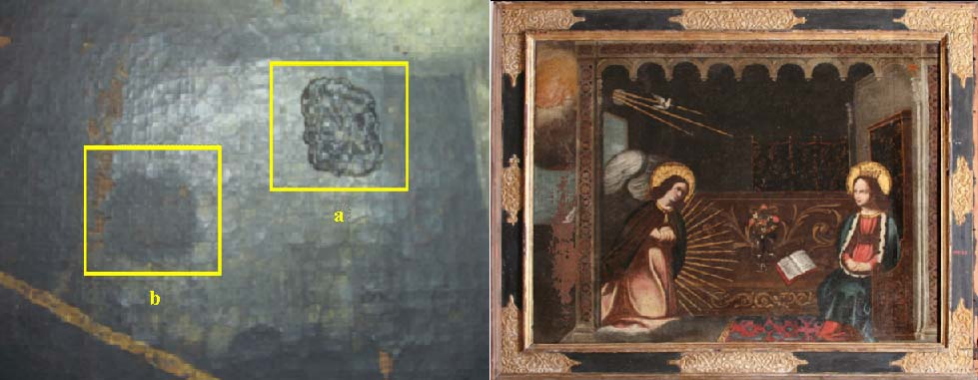
Paint art-work “*Annunciazione*” Century XVII. Regional Gallery of Sicily, Palazzo Abatellis - Palermo (Italy). Left, detail of the art-work: (a) applied gel and (b) removed gel; right, art-work after the cleaning process. From Ref. [9].

An example of the application of a surfactant system based on block copolymer and ligroin (a hydrocarbon mixture) [9] on a art-work is illustrated in Figure 1, which shows the efficiency of the gel in removing the varnish.

The surfactant systems are successfully applied to the remediation of soil and water basins contaminated by non-aqueous phase liquids (NAPLs). They are used in the *Surfactant Enhanced Aquifer Remediation* technology for their high ability to decrease the surface tension, to solubilize and mobilize the contaminant [10,11]. Successful laboratory as well as pilot-scale demonstrations tests [12] have consolidated this technology.

The study of block copolymers in colloidal systems for controlled drug delivery started around the mid-80s. Block copolymers micelles may optimize drug performance as they dissociate very slowly into the corresponding monomers allowing a longer retention of the drug and a higher concentration of the drug in the selected area. Block copolymers based on ethylene oxide and propylene oxides, were used for the synthesis of oil in water microemulsions to extract bupivacaine, which is a local anesthetic commonly used in surgical treatment, from a saline solution [13]; its overdoses can cause cardiac arrest and even the death of the patient. It was shown that the high interfacial area of the microemulsions, due to the presence of oil droplets, favors very efficiently the drug encapsulation. Being biocompatible, they were used in the pharmacological area to deliver the active principles sparingly soluble in water.

## 2. Calorimetry: A Powerful Technique to Study Physico-Chemical Processes

In the last two decades chemical thermodynamics [14] has been remarkably applied to different fields like life and material science, colloidal systems, drug delivery, chemical engineering, biopharmaceutical formulations, etc. Continuous improvements have been performed on both the experimental and the theoretical view-points and novel techniques have been designed and sophisticated models have been proposed accordingly. Theoretical approaches applied to the accurate experimental data enable the extraction of information at a molecular level.

Within this area, calorimetry has been revealed as one of the most important and relevant techniques, being sensitive even to rather weak interactions and thus the information provided may be unique. Instruments may cover a large range of temperature and pressure [14] and analyze samples in different states (gases, liquids, solids). Isothermal calorimetry has been based on microcalorimeters presenting the disadvantage of using grams of samples and to be time consuming. Nevertheless, very precise molecular insights have been obtained from the experimental data. Sophisticated isothermal titration calorimeters have been produced; they have a good performance because the measurements are less time consuming, very small amounts of samples are required and very fast data analyses can be performed. However, even in the presence of versatile apparatus expertise in the calorimetric field is a necessary condition to obtain reliable data for drawing meaningful insights.

## 3. Thermodynamics of Surfactants Micellization Derived from Enthalpy

It is well known that micellar aggregates are formed as a consequence of the tendency of hydrophobic tails to escape from water and of the polar heads to maximize their contact with water. Therefore, the transferring of the surfactant from water to the micelles involves the desolvation of the hydrophobic moiety and the variation of the hydration state of polar heads including the counterions in the case of ionic surfactants. Therefore, depending on the nature of the surfactant, the enthalpy of micellization (ΔH°_m_) may be or not the driving force of the aggregation process.

Basically two approaches have been used in the literature to evaluate ΔH°_m_. The first one [15–20] is based on the determination of the critical micellar concentration (CMC) as a function of temperature (T). The standard free energy (ΔG°_m_) is correlated to the CMC and using the van’t Hoff approach ΔH°_m_ is computed. The second method is based on direct enthalpy determinations [1–3,15,21–31] from which not only ΔH°_m_ but also the enthalpies of the surfactant in the aqueous and the micellar phases are determined. In the following, both methodologies will be described in detail.

### 3.1. The van’t Hoff Approach for Determining the Enthalpy of Micellization

**Figure 2.**
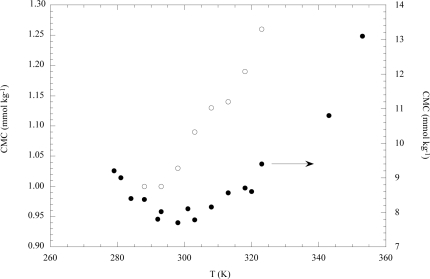
Dependence of the CMC on temperature for the aqueous solutions of sodium dodecylsulfate (●) and cetylpyridinium chloride (**○**). Data are from Ref. [15].

Usually, homologous series of cationic, anionic and non-ionic surfactants show concave trends of the CMC vs T plots with minima localized at lower temperatures the longer the alkyl chain length is. Examples [15] dealing with sodium dodecylsulfate (NaDS) and cethylpyridinium chloride in water (CPyC) are shown in Figure 2.

According to the pseudo-phase transition model [16], for an ionic surfactant the standard free energy of micellization is given by:

(1) $$\Delta \text{G}{{}^{\circ}}_{\text{m}}=\text{RT}\;(1+\alpha )\;\text{ln}\;\text{CMC}$$

where α is the degree of dissociation of the micelles. For non-ionic surfactants α=0 whereas in some cases, for 1:1 ionic surfactants α is stated unitary [17].

The ΔH°_m_ value is calculated by using the Gibbs-Helmotz equation:

(2) $$\Delta \text{H}{{}^{\circ}}_{\text{m}}=[\delta (\Delta \text{G}{{}^{\circ}}_{\text{m}}/\text{T})/\delta ({\text{T}}^{-1})]$$

The CMC dependence on temperature is often [17–20] expressed as a symmetrical parabolic curve according to Equation 3:

(3) $$\text{ln}\text{CMC}=\text{a}+\text{bT}+{\text{cT}}^{2}$$

where a, b and c are fitting parameters. By combining Eqs. 1–3, ΔH°_m_ is calculated.

Indeed, from the thermodynamic view-point, the dependence of ΔG°_m_ on temperature is given by:

(4) $$\Delta \text{G}{{}^{\circ}}_{\text{m},\text{T}}/\text{T}=\Delta \text{G}{{}^{\circ}}_{\text{m},\text{T1}}/{\text{T}}_{1}+(\Delta \text{H}{{}^{\circ}}_{\text{m},\text{T1}}/{\text{T}}_{1}-\Delta \text{Cp}{{}^{\circ}}_{\text{m}}{\text{T}}_{1})({\text{T}}^{-1}-{\text{T}}_{1}^{-1})-\Delta \text{Cp}{{}^{\circ}}_{\text{m}}\text{ln}(\text{T}/{\text{T}}_{1})$$

provided that the temperature effect on ΔH°_m_ is considered according to Equation 5:

(5) $$\Delta \text{H}{{}^{\circ}}_{\text{m},\text{T}}=\Delta \text{H}{{}^{\circ}}_{\text{m},\text{T1}}+\Delta \text{Cp}{{}^{\circ}}_{\text{m}}(\text{T}-{\text{T}}_{1})$$

where T and T_1_ represent a generic and fixed temperature, respectively, while ΔCp°_m_ is the heat capacity of micellization.

If it is assumed that ΔCp°_m_ is null, Equation 4 becomes:

(6) $$\Delta \text{G}{{}^{\circ}}_{\text{m},\text{T}}/\text{T}=\Delta \text{H}{{}^{\circ}}_{\text{m},\text{T1}}+(\Delta \text{G}{{}^{\circ}}_{\text{m},\text{T1}}/{\text{T}}_{1}-\Delta \text{H}{{}^{\circ}}_{\text{m},\text{T1}}/{\text{T}}_{1})\;\text{T}$$

Usually, the ΔH°_m_ values obtained from Equation 2 are different from those determined from the isothermal calorimetry. Such a method was indeed questioned several years ago by Holtzer and Holtzer who ascribed the limit of this approach to neglecting the effect of the variation of the aggregation number and the degree of dissociation of ionic surfactants with temperature [21]. Apart from these explanations, other factors should be considered: 1) the dependence of the CMC on temperature is usually rather small; 2) the CMC is an extrapolated property; 3) the pseudo-phase transition model is relatively approximated; and 4) the heat capacity of micellization is assumed to be null.

### 3.2. Enthalpy of Micellization from Isothermal Calorimetry

The literature ΔH°_m_ values for conventional surfactants are usually obtained from isothermal experiments like enthalpy of dilution [22–24], solution [23,25,26] or titration [1–3,15]. The enthalpy of dilution (ΔH_d_) allows to calculate the apparent molar relative enthalpies (L_Φ_) as functions of the surfactant molality (m_S_). Briefly [24]:

(7) $$\Delta {\text{H}}_{\text{d}}={\text{L}}_{\Phi }({\text{m}}_{\text{S},\text{f}})-{\text{L}}_{\Phi }({\text{m}}_{\text{S},\text{i}})$$

where L_Φ_ (m_S,f_) and L_Φ_ (m_S,i_) refer to the final and the initial states of the dilution process, respectively.

The surfactant in the pre-micellar region is described as:

(8a) $${\text{L}}_{\Phi }={\text{A}}_{\text{DH}}({\text{m}}_{\text{S}}{)}^{1/2}+{\text{Bm}}_{\text{S}}+\text{C}({\text{m}}_{\text{S}}{)}^{3/2}\ldots \;(1:1\;\text{ionic}\;\text{surfactant})$$

(8b) $${\text{L}}_{\Phi }={\text{Bm}}_{\text{S}}+\text{C}({\text{m}}_{\text{S}}{)}^{2}+\ldots \;(\text{non-ionic}\;\text{surfactant})$$

where A_DH_ is the Debye-Hückel parameter while B and C are the pair and triplet interaction parameters, respectively. By applying Eqs. 7 and 8 to the ΔH_d_ data in the pre-micellar domain, the B and C parameters (being known A_DH_ for ionic surfactants) can be determined and therefore L_Φ_ can be computed. The procedure to calculate the L_Φ_ values in the micellar region is more complex as detailed in the following. Briefly, some experiments are carried out in such a way that the final m_S_ values are lower than the CMC and the initial m_S_ values are larger than the CMC. Thus, for a given dilution process the L_Φ_ (m_S,f_) is calculated by means of Equation 8 and L_Φ_ (m_S,i_) through Equation 7. Note that this approach was also applied to the surfactant-surfactant mixtures [27].

**Figure 3.**
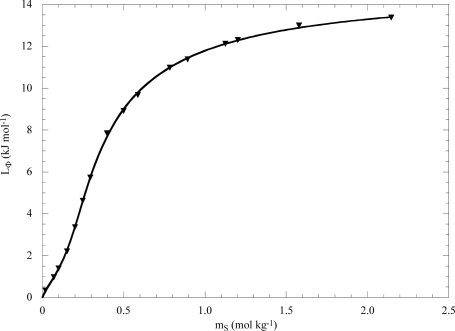
Apparent molar relative enthalpies of *N,N,N*-octylpentyldimethylammonium chloride in water as functions of the surfactant concentration at 298 K. The line is the best fit according to Eqs. 10–12. Data are from Ref. [24].

An example of L_Φ_ vs m_S_ trend for the aqueous *N,N,N*-octylpentyldimethylammonium chloride (OPC) solutions is illustrated in Figure 3. As can be seen, according to Equation 8a L_Φ_ does not change linearly to the CMC (ca. 0.15 mol kg^−1^) thereafter it sharply increases, due to the micellization process, tending to a constant value at high concentration. In the case of long alkyl chain surfactants, a smooth maximum after the CMC appears reflecting the micelle-micelle interactions which are relevant for ionic surfactants and negligible for non-ionic surfactants.

A different way [23] to calculate L_Φ_ is based on the enthalpy of solution of the surfactant in water (ΔH_s_) according to:

(9) $$\Delta {\text{H}}_{\text{s}}=\Delta \text{H}{{}^{\circ}}_{\text{s}}+{\text{L}}_{\Phi }$$

where ΔH°_s_ is the standard property.

**Figure 4.**
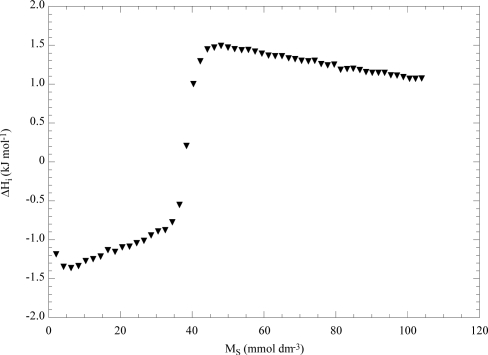
Enthalpy per moles of injected micellized surfactant (0.6 mmol dm^−3^) in water as a function of sodium decylsulfate molarity at 293 K. Data are from Ref. [28].

Most of the recent data are based on isothermal titration calorimetry (ITC) experiments [1–3,15] that are carried out by titrating a surfactant solution into water and measuring the enthalpy per moles of injectant (ΔH_i_). Figure 4 shows the dependence of ΔH_i_ on the surfactant molarity (M_S_) for the aqueous sodium decylsulfate (NaDeS) solution [28]. As can be seen, ΔH_i_ exhibits a sharp increase at the CMC (35 mM) and a small decrease in the micellar region. It is noteworthy that the graphs in Figures 3 and 4 are similar and differ in evidencing the micelle-micelle interactions which are observed only for NaDeS.

Modelling the enthalpy data is necessary to extract insights on the monomer-monomer and monomer-micelle interactions. Whatever is the model used (pseudo-phase transition [16] or mass action [23,29–31]) at a given temperature for L_Φ_ one may write:

(10) $${\text{L}}_{\Phi }=(1-{\text{X}}_{\text{M}})\;{\text{L}}_{\text{m}}+{\text{X}}_{\text{M}}{\text{L}}_{\text{M}}$$

where L_m_ and L_M_ are the partial molar relative enthalpies of the surfactant in the dispersed and the micellar states, respectively; note that (L_M_ − L_m_) corresponds to ΔH°_m_.

X_M_ is the fraction of the micellized surfactant given by:

(11) $${\text{X}}_{\text{M}}=({\text{m}}_{\text{S}}-[\text{m}])/{\text{m}}_{\text{S}}$$

where [m] is the concentration of the monomeric surfactant. In the case of the pseudo-phase transition model, [m] = CMC and ΔH°_m_ is calculated as difference between the extrapolated values at the CMC of the L_Φ_ vs m_S_ trends above and below the CMC. It may occur that, for ionic surfactants, the presence of maxima in the L_Φ_ vs m_S_ curves renders not accurate the evaluation of ΔH°_m_.

If one assumes the mass action model [30] based on a one-step aggregation process, the equilibrium constant (K_m_) for micellization is given by:

(12) $${\text{K}}_{\text{m}}=({\text{m}}_{\text{S}}-[\text{m}])/\text{N}[\text{m}{]}^{\text{N}}$$

where N is the aggregation number.

The fit of L_Φ_ vs m_S_ trend by means of Eqs. 10–12 allows calculation not only of B, C and L_M_, but also of K_m_ and N values. As an example we analyzed the L_Φ_ data for OPC in water (the fit is shown in Figure 3) and the following values were obtained: B = − 0.49±0.11 mol^−3/2^ kg^1/2^, C = 25.4±0.3 mol^−5/2^ kg^3/2^, L_M_ = 14.78±0.04 kJ mol^−1^, K_m_ = 302±30 kg^N−1^ mol^1−N^ and N = 7.7.

### 3.3. Enthalpies of Micellization of Conventional Surfactants in Water: State of Art

The ΔH°_m_ values [15] of some surfactants [decyltrimethylammonium bromide (DeTAB), sodium 1,4-bis(2-ethylhexyl)sulfosuccinate (AOT), NaDS, CPyC] obtained from both the van’t Hoff and the isothermal methods together with the CMC values are reported in Table 1. As can be seen, the CMC obtained from the different techniques are comparable while the ΔH°_m_ values differ not only in the magnitude but sometime even in the sign. This means that ambiguous insights on the driving forces of the micellization process can be drawn. Therefore, one may conclude that Equation 4 is a basic equation independent of models so that, according to Desnoyers and Perron [32], its primary use should consist in predicting the temperature dependence of ΔG°_m_. A correct calculation requires that both ΔH°_m_ and ΔCp°_m_ are obtained from direct measurements and are correctly evaluated.

**Figure 5.**
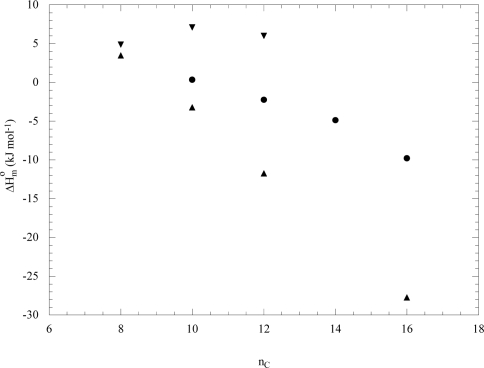
Enthalpy of micellization of alkyltrimethylammonium bromides (●, from Ref. [22]), alkylmethylpiperidinium chlorides (▾, from Ref. [33]) and alkanediyl-α,ω-bis(alkyldimethylammonium bromides) (▴, from Ref. [1]) as a function of the number of carbon atoms in the surfactant alkyl chain at 298 K.

Based on the above arguments, in the following the ΔH°_m_ values, obtained from calorimetry, will be discussed. Several investigations on homologues series of surfactants, where both the hydrophobicity and the polar head nature were systematically changed, are known. As a general feature, the ΔH°_m_ dependence on the surfactant alkyl chain length is a function of the polar head. In particular, it decreases for alkyltrimethylammonium bromides [22] (Figure 5) and it is nearly constant for sodium alkylsulfates [25]. It presents maxima in the case of alkyldimethylamine oxides [30] and alkylmethylpiperidinium chlorides [33] (Figure 5); similar results were observed by lengthening the second tail of the surfactant belonging to the *N,N,N*-alkyloctyldimethylammonium chloride series [24]. The hydrophilic head nature influences ΔH°_m_ and generally the non-ionic surfactants exhibit positive and larger ΔH°_m_ values than the ionic surfactants having the same tail size. Moreover, at a fixed alkyl chain the ΔH°_m_ decreasing by rendering more polar the head group [34].

As concerns the family of gemini surfactants [1–4], for the series of (oligooxa)-alkanediyl-α,ω-bis(dimethyldodecylammonium bromide) (12-EO*x*-12) where 0≤x≤3 [4], the ΔH°_m_ values increase with the number of the ethylene oxide (EO) groups in the spacer. Such findings were interpreted by invoking: 1) the transferring of the spacer from the aqueous phase to the micelle; 2) the folding of the spacer into the micelle; and 3) the steric hindrance between the alkyl chains. Other cationic symmetric gemini surfactants, [C_m_H_2m+1_(CH_3_)_2_N(CH_2_)_6_N-(CH_3_)_2_C_m_H_2m+1_]Br_2_ with m variable (m=7–12, 16) were investigated [1]. The CMC decreases in a no-linear manner with the alkyl chain length in agreement with the enhanced hydrophobicity. Interestingly, the ΔH°_m_ values of the surfactants with even numbered alkyl chains changed from endothermic to exothermic (Figure 5) while those of ΔH°_m_ with odd numbered alkyl chains were always endothermic assuming larger values with increasing m. This finding was ascribed to the diverse orientation of the C-C bond linked to the quaternary ammonium group between the even and the odd alkyl chains [1]. The dissymmetric cationic gemini surfactants [C_m_H_2m+1_(CH_3_)_2_N(CH_2_)_6_N(CH_3_)_2_C_n_H_2n+1_]Br_2_ with constant (n + m) value and n=6, 8, 10, 11, 12 [2], exhibit ΔH°_m_ values which decrease with the m/n ratio ascribed to the large increase of the hydrophobic contribution to micellization. Systematic studies on the effect of counterions on the micellization of C_12_H_25_(CH_3_)_2_N(CH_2_)_6_N-(CH_3_)_2_C_12_H_25_]X_2_ with X = F^−^, Cl^−^, Br^−^, Ac^−^, NO_3_^−^ showed that ΔH°_m_ values are negative due to the changes of hydration upon the association [3].

## 4. Thermodynamics of Polymeric Surfactants Micellization Derived from Enthalpy

Block copolymers are macromolecules built with monomers having different properties. They may have a variety of molecular architectures depending on the polymerization process. Among them, the di-block and the tri-block copolymers have to be mentioned [5]. Each macromolecule in solution assumes a conformation which depends on the balance between the polymer segment-segment interactions and those due to the segment-solvent interactions. In addition, the macromolecule size plays a key role within this aspect. For a block copolymer, this balance is rather complex because the constituent blocks exhibit a selective affinity to the solvent generating an amphiphilic characteristics which causes the macromolecule self-aggregation. This behaviour was observed for a variety of block copolymers in the aqueous [35,36] and the non-aqueous [37] media. The self-assembled structures (micelles of various shapes and mesophases) are similar to those formed by conventional surfactants but they may be modulated by varying the nature of monomers and the length of the segments. The polar group frequently present in the block copolymers is the poly(ethylene oxide) (PEO) which may be linked to different apolar blocks. The high solubility of PEO in water, due to its favourable penetration of oxyethylene monomer in the water structure, is changed by varying the temperature in such a way the self-assembling process can be controlled not only by concentration but also by temperature.

### 4.1. Enthalpy of Micellization

Among the block copolymers composed of PEO blocks, there are the tri-block poly(ethylene oxide)poly(propylene oxide)poly(ethylene oxide) copolymers which are denoted as EO_a_PO_b_EO_a_ where a and b are the number of the repetitive units of the ethylene oxide (EO) and the propylene oxide (PO), respectively. These copolymers are commercially available under the trade name Pluronics, have a low cost and are biocompatible [5].

The number of enthalpic studies devoted to the aggregation of block copolymers in water is quite reduced and, most of them deal with Pluronics. The methodology more commonly used to determine ΔH°_m_ is essentially based on the van’t Hoff method.

**Figure 6.**
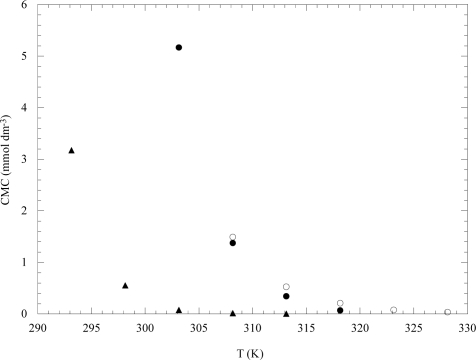
The CMC dependence on temperature of EO_13_PO_30_EO_13_ (●), EO_103_PO_39_EO_103_ (○) and EO_97_PO_69_EO_97_ (▴) in water. Data are from Ref. [38].

Examples of the plots of CMC *vs* T for the aqueous solutions of EO_13_PO_30_EO_13_ (L64), EO_103_PO_39_EO_103_ (F88) and EO_97_PO_69_EO_97_ (F127) in water are shown in Figure 6. It can be observed a large temperature sensitivity of the CMC in agreement with the high and positive ΔH°_m_ value the order of magnitude of which is hundreds of kJoules per mol.

Alexandridis *et al*. [38] performed systematic studies based, on the van’Hoff method, from which the following insights were drawn: 1) whatever is the composition and the structure of the copolymer, ΔH°_m_ is always positive making enthalpically unfavorable the process; 2) ΔH°_m_ normalized for (2a+b) is independent of the EO/PO ratio indicating that the heat involved in the aggregation does not depend on the molecular weight; 3) ΔH°_m_ for relatively hydrophobic copolymers is much larger than that of the hydrophilic ones; 4) ΔH°_m_ tends to a null value when the PO/EO ratio is close to zero indicating that the desolvation of the PO segments controls the micellization. As far we know, very few are the data from ITC [39]. The enthalpy per injection as a function of F127 concentration shows a wide inflection point ascribable to the CMC [39]. This result is opposite to that usually observed for a non-ionic surfactant because the tri-block copolymers probably contain the impurities like PEO, PPO, other di- and tri-block copolymers and furthermore they are not monodisperse.

Some ΔH°_m_ values were determined from the differential scanning calorimetry [40]. In such cases, when comparisons are possible [38,40], the ΔH°_m_ magnitude values differ from those obtained from the van’Hoff method.

**Figure 7.**
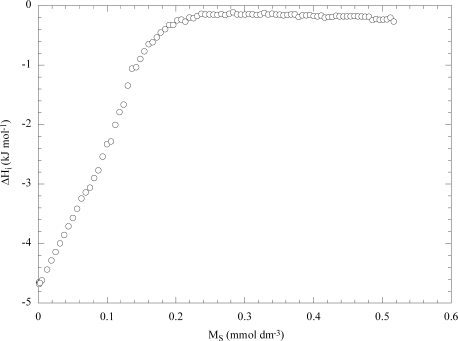
Enthalpy per injection for the water+EO_45_S_10_ mixture as a function of the copolymer concentration at 303 K. The CMC is 3×10^−3^ mmol dm^−3^. Data are from Ref. [41].

There is the family of di-block copolymers composed of EO units and styrene oxide (EO_a_S_b_ being a and b the repetitive number of the EO and the styrene units, respectively) which exhibit an enthalpic behavior [41] very different from that of Pluronics. The CMC is nearly insensitive to temperature allowing ΔH°_m_ values close to zero [41]. An example of ITC data for the aqueous solution of EO_45_S_10_ is illustrated in Figure 7. From these data, the ΔH°_m_ value of 4.5 kJ mol^−1^ is obtained that is much smaller than that evaluated for a Pluronic having comparable size; for instance, ΔH°_m_ of L64 in water obtained from DSC is 123.8 kJ mol^−1^ [40].

For the family of EO_a_S_b_, whatever the used methods are (van’t Hoff or ITC), the ΔH°_m_ decreases with the hydrophobic block tending to a null value for b=7. This effect was attributed to the reduced interactions with water as the hydrophobic styrene block was coiled in the unimeric state. Moreover, as already observed for conventional surfactants, the ΔH°_m_ values obtained from the van’Hoff equation [42] do not quantitatively agree with those derived from ITC. A similar behavior was observed for the di-block and the tri-block copolymers based on EO and phenyl glycidyl ether units [43,44]. In fact, the CMC weakly depends on temperature and provides small ΔH°_m_ values. Also, for the (ethylene oxide)_67_(phenyl glycidyl ether)_5_ it is still observed the difference between ΔH°_m_ evaluated from van’Hoff method (10 kJ mol^−1^) and from ITC (≈1.3 kJ mol^−1^). Small ΔH°_m_ values were also determined for tri-block copolymers based on PEO and phenyl glycidyl ether block ether [43]. This feature was ascribed to the hydrophobic phenyl glycidyl ether unit which is tightly coiled in the monomeric state.

## 5. Thermodynamics of Surfactant + Block Copolymer Aggregation Derived from Enthalpy

Isothermal calorimetry has provided a significant input to the comprehension of interactions between block copolymer and conventional surfactant in the aqueous phase. In the last two decades ITC studies on the aqueous copolymer+conventional surfactant mixtures were carried out. The ITC experiments are performed [39] by injecting a concentrated surfactant solution (usually well above the CMC) into a sample cell filled with a copolymer solution of known concentration. From a certain number of injections the enthalpy per mole of surfactant injected (ΔH_i_) into the copolymer solution is determined. By comparing these results with those in the absence of the macromolecule, information on the binding process between the surfactant (monomeric and/or micellized) and the copolymer are drawn. Another function largely determined is the enthalpy of transfer of the copolymer (ΔH_t_) from water to the aqueous surfactant solutions. It is evaluated as the difference between the thermal effect due to the mixing process of the solutions of copolymer and surfactant and that due to the dilution process of the same surfactant solution with water. By correcting the enthalpic effect for the dilution of the copolymer, ΔH_t_ is calculated [45–49]. The corresponding heat capacity of transfer of the copolymer (ΔCp_t_) is determined from direct measurements making reliable the obtained property [46,50,51].

### 5.1. Enthalpies of Injection

In a quite recent review, Tam *et al*. [52] devoted a noteworthy attention to ITC applied to triblock copolymer-surfactant systems. The most investigated aqueous mixtures are composed of PEO-PPO-PEO or PPO-PEO-PPO [53,54] and anionic [39,54–59], cationic [54,56,58,60,61] and non-ionic [54,59,62–64] surfactants. The new family of gemini surfactants, [C_m_H_2m+1_(CH_3_)_2_N^+^-(CH_2_)_s_-N^+^(CH_3_)_2_-C_m_H_2m+1_·2Br^−^], with variable alkyl chain length (m) and spacer (s), was also studied [65]. As a general feature, the copolymer binds significantly the surfactant. Although the literature ITC data differ in the copolymer/surfactant concentration or temperature, some general considerations can be done. The experiments performed in the presence of the unassociated block copolymer generate titration curves which have been described in terms of: 1) critical aggregation concentration which represents the onset of the surfactant binding to the copolymer (CAC); 2) saturation concentration at which the copolymer is saturated with the surfactant molecules (C_sat_); and 3) critical concentration at which the free surfactant starts to micellize.

When the copolymer is preferentially in the micellar state a more complex situation takes place. In the surfactant pre-micellar region, the titration curve does not approach that of the surfactant dilution and generally exhibits a marked exothermic peak before merging with the dilution curve in water at a large M_S_. Under these conditions, copolymer-rich/surfactant mixed micelles are formed and upon increasing the surfactant concentration they disappear on behalf of smaller surfactant-rich mixed aggregates until to the complete copolymer disaggregation.

A few investigations deal with copolymers different from Pluronics. In particular, the EO_a_S_b_ diblock copolymers in the presence of various sodium alkylsulfates [28,66] were studied. As a general feature, the addition of monomeric surfactant to the copolymer solution generates heat effects which are enhanced by increasing the surfactant hydrophobicity.

**Figure 8.**
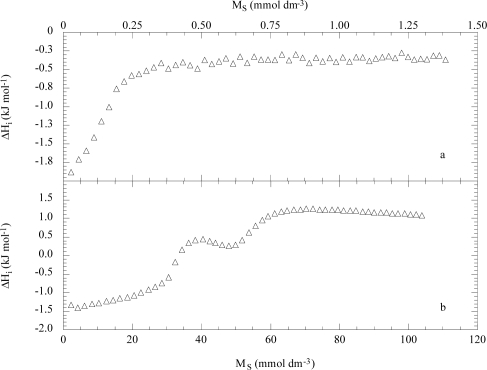
Dependence on the surfactant concentration of the enthalpy per moles of injected unmicellized 8 mmol dm^−3^ (a) and micellized 0.6 mol dm^−3^ (b) sodium decylsulfate in the presence of micellized 2.5 g dm^−3^ copolymer EO_63_S_15_ at 293 K. Data are from Ref. [28].

The NaDeS/EO_63_S_15_ system is illustrated in Figure 8a as an example. As can be seen, an exothermic enthalpic effect is registered at low M_S_ as a consequence of the interactions between NaDeS and the copolymer. In fact, NaDeS in water in the same surfactant domain presents a ΔH_i_ value constant and equal to about −0.3 kJ mol^−1^ reflecting the unimeric state of the surfactant. By titrating the same aqueous EO_63_S_15_ mixture with a micellized solution of NaDeS a more complex trend of ΔH_i_ vs m_S_ is obtained. In the region to ca. 25 mmol dm^−3^ the ΔH_i_ values are coincident with those in water (Figure 4) thereafter they deviate presenting a peculiarity with a minimum at ca. 50 mmol kg^−1^ and they merge with the values in water above at 70 mmol dm^−3^. The peculiar ΔH_i_ values were ascribed to the disruption of the surfactant-copolymer mixed micelles followed by the binding between the NaDeS aggregates and single copolymer molecules and then by the formation of free NaDeS micelles.

### 5.2. Enthalpies of Transfer

The enthalpies of transfer determined for PEO-PPO-PEO+surfactant system investigated different aspects: 1) the hydrophobicity [45,46,48–50] as well as the polar head [45,47,51] of the surfactant; 2) the copolymer molecular weight, keeping constant the EO/PO ratio, i.e., EO_76_PO_29_EO_76_ (F68), F88 and EO_132_PO_50_EO_132_ (F108); and 3) the copolymer hydrophilicity keeping constant the size of the PPO block.

**Figure 9.**
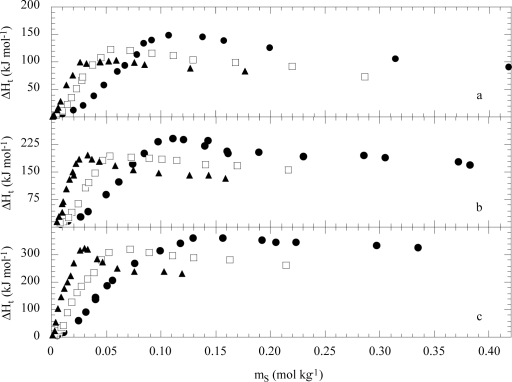
Enthalpy of transfer of copolymer from water to the aqueous sodium decanoate (●), sodium undecanoate (□) and sodium dodecanoate (▴) solutions as a function of the surfactant concentration at 298 K. a) L64; b) F68; c) F108 at 298 K. Data are from Ref. [48].

Examples of ΔH_t_ vs m_S_ are shown in Figure 9. For a given surfactant, ΔH_t_ sharply increases with m_S_ reaching a maximum and then decreases tending to a constant value. The location of the extremum is nearly independent of the nature of the macromolecule since it appears at a m_S_ value close to the CMC. By increasing the copolymer molecular weight, at fixed EO/PO ratio, ΔH_t_ becomes more endothermic. Moreover, decreasing the copolymer hydrophilicity (from F68 to L64) ΔH_t_ is less endothermic and the maximum is broader. The surfactant hydrophobicity enhances the extrema in the ΔH_t_ vs m_S_ curves and the ΔH_t_ magnitude decreases. In the presence of cationic or non-ionic surfactants the ΔH_t_ vs m_S_ curves are sigmoidal S-shaped which do not exhibit extrema near the CMC region [45].

**Figure 10.**
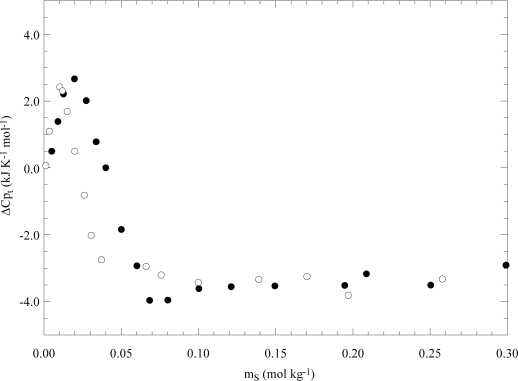
Heat capacity of transfer of L64 from water to the aqueous sodium undecanoate (●) and sodium dodecanoate (○) solutions as a function of the surfactant concentration at 298 K. Data are from Ref. [50].

The heat capacity of transfer function is a complex property being the derivative of the enthalpy of transfer with respect to temperature. For all of the investigated systems, the ΔCp_t_ vs m_S_ trend shows a maximum and a minimum. By increasing the surfactant hydrophobicity, the maximum becomes sharper and is located at lower m_S_ values whereas the minimum tends to disappear as Figure 10 illustrates. For associated copolymers [50], ΔCp_t_ decreases monotonically with m_S_ assuming negative values. The interpretation of ΔH_t_ as well as ΔCp_t_ data was successfully done by invoking the formation of copolymer-surfactant aggregates taking place in the pre- and the post-micellar regions. In the case of heat capacity [50], the relaxation terms due to the effect of temperature on the formation of these aggregates was also taken into account.

### 5.3. Modeling the Enthalpies of Aqueous Copolymer+Surfactant Mixtures

In spite of the huge amount of experimental thermodynamic data available for copolymer-surfactant mixtures, only a very few thermodynamic models have been proposed for a quantitative treatment. A model which uses an Origin based non-linear function did permit to analyze [67] ITC results by determining the CAC and the C_sat_ values and the corresponding enthalpies. This approach was also applied to several ITC data of the copolymer-surfactant systems [39,53–68].

A few years ago, De Lisi *et al*. [47] proposed a thermodynamic model aimed at describing quantitatively the thermodynamic (namely, volume, enthalpy, heat capacity) properties of transfer of the unimeric copolymer, at fixed concentration, from water to the aqueous surfactant solutions at variable composition. The approach stated that: 1) in the pre-micellar region the surfactant molecules interact with the copolymer to form surfactant-copolymer aggregates; and 2) once that the CMC is reached, the surfactant undergoes the micellization and therefore the copolymer may be incorporated into the micelles forming mixed aggregates. By applying such a model to the enthalpy of transfer the following equation was obtained [47]:

(13) $$\Delta {\text{H}}_{\text{t}}=2{\text{B}}_{\text{PS}}x_{\text{P}}[\text{m}]+x_{\text{C}}\Delta {\text{H}}_{\text{C}}+\{([{\text{m}}_{0}]-[\text{m}]-\text{j }x_{\text{C}}\;{\text{m}}_{\text{P}})/{\text{m}}_{\text{P}}\}\;\Delta {{\text{H}}^{{}^{\circ}}}_{\text{m}}+x_{\text{M}}\Delta {\text{H}}_{\text{M}}$$

where B_PS_ stands for the copolymer-surfactant interaction parameter in the aqueous phase, *x*_P_ is the fraction of the dispersed copolymer whereas [m_0_] and [m] represent the monomer surfactant concentration in the absence and the presence of the copolymer, respectively. *x*_C_ is the fraction of the surfactant-copolymer pre-micellar aggregates, which is expressed in terms of the equilibrium constant (K_C_) and the j stoichiometry of the aggregate, and ΔH_C_ is the enthalpy change for the formation of these aggregates; *x*_M_ is the fraction of the copolymer-micelle aggregates, which is related to the equilibrium constant (K_M_) and the w stoichiometry of the aggregate, and ΔH_M_ is the enthalpy change for the copolymer-micelle aggregates formation. The K_C_, j and ΔH_C_ parameters are provided by the fit of data in the pre-micellar domain while K_M_, w and ΔH_M_ by fitting the data in the micellar region.

**Figure 11.**
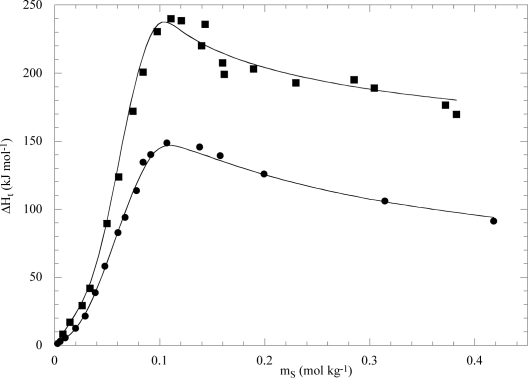
Enthalpies of transfer of unimeric F68 (■) and L64 (●) from water to the aqueous sodium decanoate at 298 K. Lines are best fits according to Equation 13. Data are from refs. [46,48].

Figure 11 shows a few examples of the minimizing procedure according to Equation 13. The validity of this model was proved [46,47] and even confirmed by independent small angle neutron scattering (SANS) studies [69].

Another model aimed at interpreting the ITC data [54] is based on the following processes: 1) the binding of monomeric surfactant to sites of the copolymer; 2) the interactions between a certain number of surfactant monomers and the copolymer sites forming a mixed aggregate; and 3) the formation of free micelles with a given aggregation number. A good simulation of the enthalpy data for the NaDeS+PO_19_EO_33_PO_19_ was obtained according to this approach.

### 5.4. Quantitative Insights on the Surfactant-Copolymer Microstructures

Analyzing the enthalpies of transfer, by means of the De Lisi *et al*. model, is powerful because from the fitting parameters one may calculate the standard free energy (ΔG°_i_) and entropy (TΔS°_i_) for the formation of copolymer-surfactant aggregates according to Equation 14:

(14) $$\Delta \text{G}{{}^{\circ}}_{\text{i}}=-\text{RT}\;\text{ln}\;{\text{K}}_{\text{i}}\;\text{T}\Delta \text{S}{{}^{\circ}}_{\text{i}}=\Delta {\text{H}}_{\text{i}}-\Delta \text{G}{{}^{\circ}}_{\text{i}}$$

where the subscript i is replaced by C and M for the copolymer-surfactant pre-micellar and micellar aggregates, respectively. Furthermore, the minimizing procedure provides the stoichiometries of the mixed aggregates which, whenever comparisons are possible, well agree with those provided by SANS studies [69]. In the following, the main results dealing with Pluronic+surfactant systems will be illustrated.

### 5.5. Formation of Surfactant-Copolymer Pre-Micellar Aggregates

Detailed insights on the interactions between the homologues series of sodium alkanoates [48,49] and some block copolymers, i.e. L64, F68, F88 and F108 are available.

To take into account for the different amounts of EO and PO units in the various copolymers, the properties for the surfactant-copolymer pre-micellar aggregates were calculated as ΔY_C_/(2a+b) (remember that a and b are the number of repetitive units of EO and PO, respectively).

**Figure 12.**
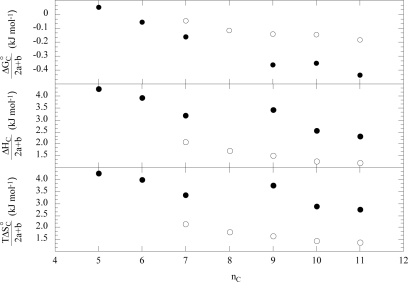
Standard free energy, enthalpy and entropy for the formation of copolymer+surfactant pre-micellar aggregates, normalized for (2a+b), as functions of the number of carbon atoms in the sodium alkanoates alkyl chain at 298 K. ●, L64; ○, F68. Data are from Ref. [49].

The increasing copolymer molecular weight slightly moves the dependence of ΔG°_C_/(2a+b) on the surfactant alkyl chain toward less negative values. The ΔG°_C_/(2a+b) for L64 is lower than that for F68 that is consistent with the L64 larger hydrophobicity (Figure 12). The enthalpy and the entropy data normalized for (2a+b) superimposed to each others for F68, F88 and F108 and smaller than the corresponding properties for L64 (Figure 12).

The high and the positive values of ΔH_C_/(2a+b) and TΔS°_C_/(2a+b) reflect the surfactant and the copolymer release water molecules from their low polar moieties which interact through the van der Waals forces forming a micro-environment similar to that of conventional micelles. The increasing of the surfactant hydrophobicity causes the entropy decrease because of the larger constraints in the conformational state of the copolymer.

Although the effect of the polar head was not extensively studied, some interesting information can be drawn. By comparing the behaviour of block copolymers in the presence of sodium undecanoate [48] and DeTAB [47] it emerges that the cationic surfactant does not form pre-micellar aggregates while the anionic one does. This unexpected result was confirmed by SANS experiments [69].

### 5.6. Formation of Surfactant-Copolymer Micellar Aggregates

**Figure 13.**
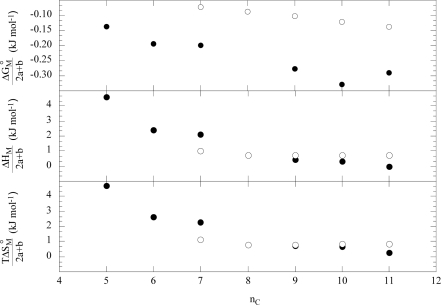
Standard free energy, enthalpy and entropy for the formation of copolymer+surfactant micellar aggregates, normalized for (2a+b), as functions of the number of carbon atoms in the sodium alkanoates alkyl chain. ●, L64; ○, F68. Data are from Ref. [49].

Whatever is the copolymer nature [49], the standard free energy (ΔG°_M_) for the formation of the copolymer-micelle aggregates is controlled by both hydrophilic and hydrophobic forces and it is governed by entropy. ΔG°_M_/(2a+b) for a given surfactant becomes more negative with decreasing the macromolecule size. The enthalpies and the entropies are rather close for F68 and F88 while they differ from those of F108. Comparing F68 and L64 behaviour (Figure 13) turns out the larger hydrophobic character of L64. As Figure 13 shows, the surfactant hydrophobicity plays a remarkable role on the enthalpy and entropy of L64 with respect to F68 indicating that the interactions between micelles and copolymer generate relevant conformational effects and degree hydration changes of the copolymer. The loss of conformational freedom was also invoked to explain the heat capacity of the copolymer+surfactant micellar aggregates [50].

As concerns the effect of the polar head of the surfactant, the octylpyridinium chloride (OPyC) micelles do not distinguish between L64 and F68 while significant differences were observed for the enthalpy and the entropy which are about twice for F68 [47]. By comparing OPyC and octyldimethylamine oxide (ODAO) it turns out that the OPyC/L64 mixed micelles are more stable than those of ODAO/L64 [47]. The DeTAB/L64 mixed aggregate is more enhanced than that of DeTAB/F68. Finally, the interactions between the polar head of DeTAB and L64 stabilize the mixed aggregate more efficiently than the polar head of sodium undecanoate [47,48].

## 6. Conclusions

This review aimed at providing a general overview of the issue of nanostructures consisting of block copolymers, conventional surfactants and their mixtures defined through the enthalpy function. The aggregation of conventional surfactants in water was analyzed to the light of the two methodologies generally used to determine the enthalpy of micellization: 1) the van’t Hoff approach based on the determination of the critical micellar concentration as a function of temperature; and 2) the isothermal calorimetric method which determines the enthalpy function (apparent molar relative enthalpy, enthalpy of injection, enthalpy of solution, etc.) as a function of the surfactant concentration. The extensive literature data allowed to evidence the limits of the van’Hoff approach compared to the isothermal calorimetry. Although the block copolymers have been studied in a less extent, the available findings allowed to draw a picture on their enthalpic behaviour essentially determined by means of the van’Hoff approach. Finally, attention was focussed on the aqueous block copolymer+surfactant mixtures described through the enthalpies of injection and the enthalpies of transfer. The literature models used to analyze the data were discussed and their relevance for obtaining information at molecular level on the copolymer/surfactant interactions was highlighted. Furthermore, in some cases, the stoichiometries of the copolymer+surfactant mixed aggregates were provided and resulted in a good agreement with information obtained from structural techniques.

## Figures and Tables

**Table 1.** The CMC and the enthalpies of micellization of some surfactants in water at 298 K.

**Surfactants**	**CMC (mM)**	**ΔH°m (van’t Hoff)**	**ΔH°m (calorimetry)**
NaDS^1^	7.75^a^;7.70^b^	−20.9	−0.81
CPyC^1^	0.96^a^;1.03^b^	−38.5	−4.5
AOT^1^	2.72^c^;2.68^b^	−4.3	3.12
DeTAB^2^	58.0^a^;57^b^	−13.0	0.89

Units are: mmol dm^−3^ for CMC; kJ mol^−1^ for ΔH°_m_;

^a^ From conductivity;

^b^ From calorimetry;

^c^ From surface tension.

^1^ From Ref. [15];

^2^ From Ref. [20].
